# Can We Predict Failure of Mitral Valve Repair?

**DOI:** 10.3390/jcm8040526

**Published:** 2019-04-17

**Authors:** Simone Gasser, Maria von Stumm, Christoph Sinning, Ulrich Schaefer, Hermann Reichenspurner, Evaldas Girdauskas

**Affiliations:** 1 Department of Cardiovascular Surgery, University Heart Center Hamburg, 20251 Hamburg, Germany; m.stumm@uke.de (M.v.S.); si.albers@uke.de (H.R.); e.girdauskas@uke.de (E.G.); 2 Department of General and Interventional Cardiology, University Heart Center Hamburg, 20251 Hamburg, Germany; c.sinning@uke.de (C.S.); schaefer.kardiologie@marienkrankenhaus.org (U.S.)

**Keywords:** mitral valve regurgitation, surgery, cardiothoracic surgery, minimally invasive mitral valve repair, redo surgery, echocardiography

## Abstract

Objective: To identify echocardiographic and surgical risk factors for failure after mitral valve repair. Methods: We identified a total of 77 consecutive patients from our institutional mitral valve surgery database who required redo mitral valve surgery due to recurrence of mitral regurgitation after primary mitral valve repair. A control group of 138 patients who had a stable echocardiographic long-term result was included based on propensity score matching. Systematic analysis of echocardiographic parameters was performed before primary surgery; after mitral valve repair and prior to redo surgery. Risk factor analysis was performed using multivariate Cox regression model. Results: Redo surgery was associated with the presence of pulmonary hypertension ≥ 50 mmHg (*p* = 0.02), a mean transmitral gradient > 5 mmHg (*p* = 0.001), left ventricular ejection fraction ≤ 45% (*p* = 0.05) before surgery and mitral regurgitation ≥moderate at time of discharge (*p* = 0.002) in the whole cohort. Patients with functional mitral valve regurgitation had a higher tendency to undergo redo surgery if preoperative left ventricular end-diastolic diameter exceeded 65 mm (*p* = 0.043) and if postoperative tenting height exceeded 6 mm (*p* = 0.018). Low ejection fraction was not significantly associated with the need for redo mitral valve surgery in the functional subgroup. Conclusions: Recurrent mitral regurgitation is still a valuable problem and is associated with relevant perioperative mortality. Patients with severe mitral regurgitation should undergo early mitral valve repair surgery as long as systolic pulmonary artery pressure is low, left ventricular ejection fraction is preserved, and LVEED is deceeds 65 mm.

## 1. Introduction

Mitral valve dysfunction (MVD) is the second most common form of heart valve disease in adults [1]. The reported annual incidence of MV disease in the industrialized nations is ~3% [1]. In general, two main pathophysiological entities of MVD exist which are completely different in terms of their treatment strategies and prognosis:

Degenerative MV is the most frequent mechanism of mitral regurgitation (MR) and leads to leaflet prolapse due to elongation or rupture of chordal apparatus or, less frequently, to restrictive leaflet motion due to calcification or inflammation. An increased motion of the free edge of one or both leaflets overriding MV annular plane during the systole is defined as leaflet prolapse (Carpentier Type II dysfunction). In contrast, patients with the type IIIa dysfunction have a restricted leaflet motion due to leaflet thickening causing scarring during both diastole and systole. 

Ischemic mitral incompetence leading to functional mitral regurgitation (FMR) is the most frequent mechanism of MR in developed countries [1]. It results from a global and/or regional left ventricular remodeling which leads to the distortion of the whole MV apparatus, including chordae, annulus, and leaflets. Ischemic MR is characterized by restrictive mitral leaflet motion during the systole due to left ventricle (LV) remodeling which occurs as a sequel of ischemic heart disease (Carpentier Type IIIb dysfunction) [1]. Patients with type I dysfunction have an extensive MV annulus dilatation and normal leaflet motion, with the free edges of the leaflets positioned 5 to 10 mm below the plane of the annulus due to annular dilatation [1].

Surgical treatment for severe mitral valve regurgitation is recommended for symptomatic patients with a left ventricular ejection fraction (LVEF) > 30% and asymptomatic patients if LVEF is 30–60% and/or left ventricular end-systolicend-systolic diameter (LVESD) exceeds 40 mm (Class 1B Recommendations) [2]. Mitral valve repair (MVR) has become a gold standard for the correction of significant degenerative mitral regurgitation (MR) in symptomatic as well as in asymptomatic patients caused by posterior leaflet prolapse in any case or for anterior/bileaflet prolapse whenever is it is expected to be durable and associated with low morbidity and mortality, and is recommended (class I) by current guidelines [2,3]. In patients suffering from FMR (or severe secondary mitral valve regurgitation) who do not require simultaneous revascularization current guidelines are much more restrictive. Surgery is indicated in patients with an LVEF ≤ 30% who remain symptomatic despite optimal medical management and have a low operative risk [4]. 

Reoperation rate due to the recurrence of MV regurgitation was recently reported 5% at 5 years and between 6.2 and 13.2% at 10 years postoperatively, not taking into account underlying valve pathology. Nondegenerative valve etiology was shown to predict death after mitral valve repair [5]. However, the echocardiographic and surgical variables associated with the recurrence of mitral regurgitation/redo mitral valve surgery after MV repair remain to be clarified. Our article aims to analyze the risk factors associated with the redo MV surgery after previous MV repair with the special focus on echocardiographic and surgical variables at the time of mitral valve repair.

## 2. Materials and Methods

### 2.1. Study Population

**Figure 1:**
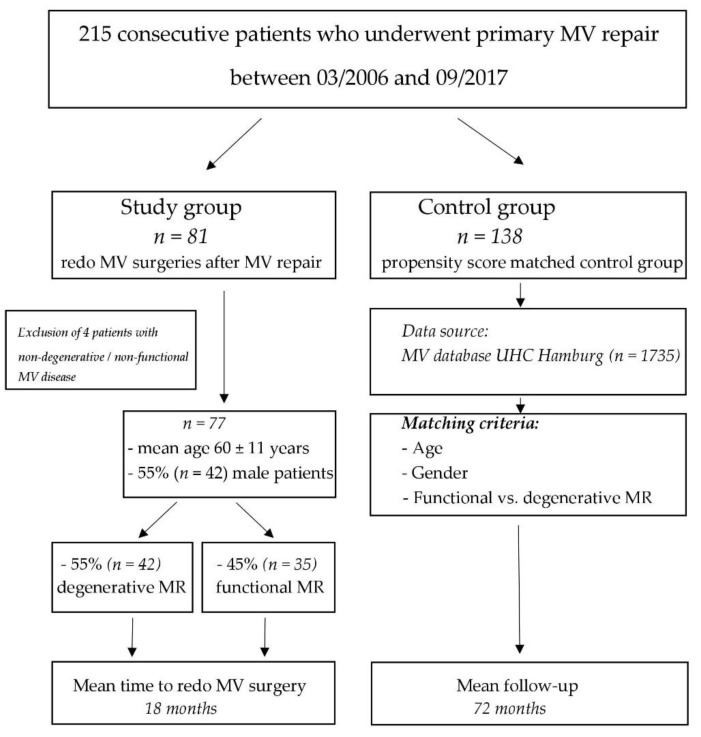
Patient enrollment flowchart. MV, mitral valve; UHZ, University Heart Center, Hamburg; MR, mitral regurgitation; SAM, systolic anterior movement.

We systematically screened our institutional MV database to identify all consecutive patients who underwent redo MV procedure after previous MV repair during the study period between March 2006 and September 2017. Inclusion criteria were degenerative or functional mitral valve regurgitation leading to MVR, and the availability of the pre- and postoperative echocardiographic images for re-review. We excluded 4 patients who had a very specific etiology of MV regurgitation, and therefore could be assigned neither to the degenerative MR nor to FMR subgroup (i.e., three patients with a cleft in the anterior mitral leaflet and one patient with systolic anterior motion (SAM) which caused MR). A total of 77 consecutive patients who underwent redo MV surgery after previous MV repair served as our study population. A total of 45 (58%) patients had their initial MV surgery at our institution, while the remaining 32 (42%) patients were treated externally. In the control group we considered those MVR patients who had a good functional outcome (i.e., residual MR ≤ 1) for at least 5 years after primary MVR (Figure 1). Propensity score based matching (3:1) was implemented and the control group was adjusted for age, gender and underlying valve pathology (i.e., degenerative vs. functional MR). All patients agreed to process surgical and echocardiographical data for research. The paper reports on retrospective research. All data analyzed were collected as part of routine diagnosis and treatment. No ethical considerations apply.

### 2.2. Echocardiographic Assessment

Transthoracic echocardiography was routinely performed before initial procedure, at hospital discharge and before redo intervention. Echocardiographic assessment was performed by an attending cardiologist at institutional echolab using a standardized protocol which characterized LV/RV function, LV end-diastolic and end-systolic diameters, left atrial (LA) volume, grade of MR, MV pathology, transmitral pressure gradients, and estimated systolic pulmonary artery pressure if tricuspid regurgitation (TR) was present. Preoperative transesophageal echo was performed to specify MV pathology before redo surgery. Valve pathology was described using the Carpentier classification. Guidelines of the European Association of Cardiovascular Imaging were used for the quantification of echordiographic findings [6]. Specifically, in FMR patients, we measured tenting parameters to quantify the severity of LV remodeling: (1) PML angle (= angle between posterior mitral leaflet (PML) and mitral annular plane in the end-systole), (2) anterior mitral leaflet (AML) length in the A2 segment and (3) tenting height (i.e., the distance between the coaptation line of mitral leaflets and the annular plane) were measured in the parasternal long-axis view.

### 2.3. Operative Technique

Surgery was performed via median sternotomy or right anterolateral mini-thoracotomy, depending on patients’ comorbidities/surgical risk and the concomitant procedures required. In degenerative mitral valve disease, leaflet prolapse was addressed first [7] using a leaflet resection or artificial neochordae implantation techniques. In Barlow’s disease bileaflet prolapse was corrected, followed by subsequent implantation of a large annuloplasty ring [8]. Circumferential ring annuloplasty was generally used in the degenerative mitral regurgitation (DMR) and ring sizing was performed according to the surface area of AML and total mitral valve orifice area. Type I MR was usually addressed by a downsized annuloplasty ring only (11). In Type IIIb MR a specifically designed annuloplasty ring (e.g., Carpentier–Edwards–McCarthy–Adams IMR ETLOGIX-Ring, Edwards Lifesciences, Irvine, CA, USA) with a reduced a–p diameter was used. Matress U-shaped sutures were used in the posterior mitral annulus to reduce the risk of tear of annuloplasty ring. Adjunct subannular techniques in Type IIIb MR were introduced more recently to restore optimal leaflet coaptation plane and to reduce the tenting area [9]. Papillary muscle repositioning is now routinely used for this purpose which has been recently published by our group [9]. Intraoperative transesophageal echocardiographic study was routinely performed to assess MV competence after discontinuation from cardiopulmonary bypass (CPB). For the present study, the definition of a minimally invasive operation was limited to MV surgery performed through a right minithoracotomy. 

### 2.4. Statistical Analysis

SPSS 24 (IBM, Armonk, NY, USA) was used for all statistical analyzes. The data are presented as mean ± standard deviation for continuous variables and as percentages for categorical variables, unless otherwise specified. Propensity score matching was performed between study and control group using 3 adjusting variables: age, gender, and etiology of MVD (i.e., degenerative vs. functional MR). Univariate analysis was performed using *t*-test for group the numeric variables and chi-squared test/Fisher exact test for the nominal variables. Cox proportional hazards regression was performed to identify risk factors predisposing patients to mitral valve redo surgery. All variables from univariate analysis with *p* < 0.1 were included in the multivariate model. *p*-values < 0.05 were considered statistically significant. 

## 3. Results

### 3.1. Baseline Characteristics

A total cohort of 215 patients was analyzed (i.e., after inclusion of the control group based on propensity score matching). The cohort consisted of degenerative MR subgroup (*n* = 147; 68%) and functional MR subgroup (*n* = 68; 32%). Mean age was 61 ± 11 years and 131 patients (61%) were male. Preoperative variables in the whole study cohort and in both study subgroups are presented in Table 1. The mean follow-up period was 72 ± 11 months. Redo mitral surgery was performed at a median interval of 18.5 months after previous mitral valve repair and resulted in an in-hospital mortality of 8.5% compared to 0.14% after primary surgery. The median interval was predominantly due to early failures in the type IIIb FMR patients’ subgroup, as opposed to the late failures in our DMR cohort. Early redo surgery war required in eight DMR patients due to endocarditis and five DMR patients referred for early redo MV surgery after primary MV repair surgery in an external institution. Increased mortality rate of 8.5% in the entire redo cohort was predominantly due to early deaths in the FMR cohort. Patients in the FMR cohort were significantly older, had much higher surgical risk scores and severely reduced systolic LV function. Contrarily, in-hospital mortality in the DMR cohort was low (1.8%) (Table 1).

### 3.2. Surgical Strategies

A minimally invasive approach via right anterolateral mini-thoracotomy was used in 133 patients (62%). An annuloplasty ring between 28 and 34 mm diameter was used in the majority of patients (i.e., *n* = 181 (84%)). The most frequently used annuloplasty ring type was Carpentier–Edwards Physio–II (Edwards Lifesciences, Irvine, CA, USA) in 166 patients (77%). A total of 50 (23%) patients underwent concomitant procedures, 13 of them with degenerative mitral valve disease. In the degenerative MR subgroup (*n* = 147), 53 (36%) patients undewent polytetrafluoroethylene (PTFE) neochordae implantation, while resection strategy was used in 75 (51%) patients. Univariate analysis showed a significant difference in strategy of treatment of prolapse for recurrence rate of MR (i.e., neochoardea implanation vs. resection; *p* = 0.004) which could not be confirmed in our multivariate model. Ring annuloplasty was shown to be necessary for success of MVR, especially for patients with degenerative mitral valve disease only (hazard ratio (HR) 4.1, *p* = 0.04). Choice of ring size made no statistically significant difference in FMR patients. In FMR patients (*n* = 67), 29% of repairs were performed through minimally invasive thoracotomy. Thirteen patients (19%) required concomitant coronary artery bypass graft (CABG) and 7 (10%) additional tricuspid valve repair. Subannular techniques were not used in the primary MV repair procedures.

### 3.3. Echocardiographic Parameters Associated with the Redo MV Surgery in the Whole Study Cohort

Multivariate Cox regression analysis revealed a statistically significant association between the redo MV surgery and preoperative systolic pulmonary artery pressure ≥ 50 mmHg (HR 2.3; *p* = 0.02), preoperative mean transmitral pressure gradient >5 mmHg (HR 3.3, *p* = 0.001), and reduced LVEF (≤45%) before surgery (HR 2.1; *p* = 0.05) regardless underlying valve pathology. AML length, left ventricular end-diastolic diameter (LVEDD), LVESD, and tricuspid regurgitation ≥moderate demonstrated no significant association with the redo MV surgery in the multivariate cox regression analysis. All of these echocardiographical parameters were collected prior to initial mitral valve repair.

Mitral regurgitation ≥moderate at the time of hospital discharge after MVR was significantly associated with the need of MV reintervention during the follow-up (HR 5.8; *p* = 0.002). The data of cox regression analysis for the whole cohort is summarized in Table 2.

### 3.4. Predictors for Redo MV Surgery in the Degenerative MR Subgroup

Univariate analysis revealed a trend towards increased risk of redo MV surgery in DMR patients with non-PML prolapse. However, there was no significant association between this variable and redo MV surgery in the multivariable Cox regression analysis. None of the pre- or postoperative echocardiographic parameters showed a significant association with the redo MV surgery, including TR ≥moderate, type of leaflet disease or LVEDD, and LVESD diameters systole or diastole, prior, and after MV repair in degenerative patients. For further details see Table 3.

### 3.5. Echocardiographic Parameters Associated with Redo MV Surgery in the FMR Subgroup

Reduced LV ejection fraction (≤45%) pre- or postsurgery was not associated with an increased risk of redo MV surgery in the FMR subgroup (Table 4). Furthermore, we were unable to demonstrate a significant association between preoperative tenting parameters (i.e., PML-angle, tenting height) and the redo MV surgery in the FMR cohort. The length of AML as well as elevated systolic pulmonary artery pressure (sPAP) in the preoperative echocardiography had similarly no significant association with MV reinterventions (Table 4). Only the preoperative LV end-diastolic diameter (LVEDD) ≥65 mm (HR 3.67; *p* = 0.043) showed an increased risk for redo surgery.

Furthermore, Cox regression analysis revealed a significant impact of postrepair tenting height on the risk of redo MV surgery. Post-MVR tenting height of ≥6 mm (HR 2.3; *p* = 0.018) had a negative impact on the risk of mitral valve redo surgery in our FMR cohort.

## 4. Discussion

The prevalence of reoperation due to recurrent MV regurgitation was recently reported 5% at 5 years and 9.5% at 10 years postoperatively. Nondegenerative valve etiology was shown to predict death after mitral valve repair [5]. We strongly consider redo MV surgery for failed repair in the FMR entity, especially in the setting of an obvious clinical deterioration and if re-repair seems possible, although clinical guidelines for this approach are very restrictive. However, the echocardiographic and surgical variables associated with the recurrence of mitral regurgitation/redo mitral valve surgery are still an object of controversial debate. 

The recurrence of MR does not only increase the long-term mortality but significantly worsens quality of life [10]. MR recurrence in DMR patients with an isolated anterior leaflet prolapse or bileaflet prolapse is of major clinical interest and warrants detailed comparison of surgical techniques and echocardiographic parameters which may lead to repair failure.

The most important findings of our current study are (1) preoperative echocardiographic parameters can help to predict success of mitral valve repair. In particular, our study revealed systolic pulmonary artery pressure ≥ 50 mmHg, a mean transmitral pressure gradient > 5 mmHg and reduced LVEF ≤ 45% as significant parameters associated with recurrence of MR leading to redo surgery in the whole population. (2) Left ventricular function had no impact on success of mitral valve repair in FMR, but left ventricular end-diastolic diameter as well as postoperative tenting height did. We imply that patients with severe MR should be admitted to surgery as early as possible and that repair is also feasible in FMR patients with poor ventricular function.

### 4.1. Whole Study Population

Our study confirms that reduced ejection fraction is detrimental for recurrent MR in the whole study population that underwent MVR and for survival of DMR patients undergoing MVR [11,12,13,14,15]. Elevated systolic pulmonary artery pressure has also been shown to have a negative impact on survival (all MV etiologies) as well as on recurrence of MR for DMR patients [11,12,15,16,17]. We could confirm that finding in our study for the whole study cohort regardless etiology of MVR. The possible pathophysiological explanation for this finding might be referral to surgery in an advanced stage of MV disease with progressed left ventricular and left atrial remodeling with complex changes not only effecting systolic but also diastolic function. SPAP improves early after surgery, but residual pulmonary hypertension was observed in all patients with pulmonary artery pressure ≥ 40 mmHg and predicted operative mortality, long-term mortality, and morbidity in Ghoreishi et al.’s study [17]. 

Another finding of our study was higher reoperation rates for patients with preoperatively elevated systolic transmitral valve gradient (i.e., mean transmitral gradient ≥ 5 mmHg). This finding is consistent with Coutinho et al.’s study on failures of MVR for degenerative mitral valve disease and might be explained by extensive MV leaflet degeneration which leads to progressive mitral valve stenosis/regurgitation after MVR [18]. 

### 4.2. Degenerative Mitral Regurgitation (DMR) Subgroup

Recently published data reported a 5.9% reoperation rate during 20 years of follow up in DMR patients [12,13,19,20]. The need for redo surgery was associated with an increased age, reduced LVEF, isolated AML prolapse, lack of annuloplasty, long CPB time, elevated sPAP, and evidence of myxomatous changes in the MV, as well as preoperative history of atrial fibrillation (AF) [12,13,19,20]. All of our DMR patients received ring annuloplasty. All remaining variables were not significantly associated with need for redo surgery in our DMR subgroup. We did not examine the impact of AF on redo surgery in our population. Localization of mitral valve prolapse had no impact on outcome in our study cohort. This might be explained by the learning curve of mitral valve surgeons over the last decades and their present experience in treating any mitral valve pathology. The studies reporting isolated AML prolapse and lack of annuloplasty as independent predictors of redo mitral valve surgery represent a rather historical cohort (24). These reports may represent the surgical evolution and technical improvements in MV repair techniques, while the results of posterior leaflet prolapse repair were initially better than those of repaired nonposterior leaflet lesions [21]. Our study supports the recent findings that bileaflet and anterior leaflet prolapse repair can be performed with similar long-term results as compared to the posterior leaflet prolapse in experienced centers [18]. 

### 4.3. Functional Mitral Regurgitation Subgroup

Preoperatively enlarged LVEDD ≥ 65 mm was associated with an increased reoperation rate in the FMR subgroup.

Increased tenting height has been previously reported as a determinant of FMR recurrence after an isolated annuloplasty [10], and was also found to be decisive for outcome in our FMR cohort (i.e., tenting height ≥ 6 mm at discharge transthoracic echocardiography). The deleterious effect of increasing LVEDD diameter has been similarly reported by the previous studies [10,22,23,24], and is supported by our subgroup analysis for FMR patients (LVEDD ≥ 65 mm). Increased PML-angle was reported to be a predictor of FMR recurrence as well as basal LV aneurysm/dyskinesia, which could not be confirmed [24]. The possible explanation for that is our limited sample size and potential heterogeneity of FMR cohort including ischemic, dilated, and valvular cardiomyopathies, and that basal LV aneurysms/dyskinesia were not captured by our MV database. 

There is sufficient amount of evidence in the literature showing that an isolated annuloplasty leaves uncorrected residual mitral leaflet tethering and therefore results in high MR recurrence rates in type IIIb FMR disease [25]. MR recurrence rates of 28 to 58% have been reported during the first 1–2 postoperative years after an isolated annuloplasty in type IIIb FMR [26,27,28]. Such dismal results of an isolated annuloplasty in type IIIb FMR disease underscore the need for additional subannular maneuvers to address the distorted LV geometry in those patients. Given the fact that mitral leaflet tethering in type IIIb FMR results from progressively increasing distance between the tips of papillary muscles and the mitral annular plane, an isolated annuloplasty that addresses ‘only’ mitral valve annular plane is unable to pathophysiologically correct the underlying leaflet tethering [26]. Therefore, papillary muscle repositioning (supplementary video S1) added to the standard ring annuloplasty procedure, has a potential to reduce late recurrence rate of type IIIb MR and thereby eliminate the reoperation risk [29,30,31,32]. 

## 5. Study Limitations

Our study has some important limitations. Although propensity score matching was intended to overcome the limitations of this retrospective analysis, there may still be some important differences between the study groups. The inclusion of patients with different MV pathologies in the analysis (i.e., degenerative and functional MR) may potentially weaken the findings of our study. However, the sample size in both MR subgroups was limited and the combination of both resulted in a reasonable size of the total study cohort. Experience in mitral valve repair possibly varied between surgeons performing primary mitral valve repair. Including various surgeons from different institutions is a limitation of our study. Another major limitation is that our study endpoint of redo MV surgery does not necessarily identifies all patients with the recurrent relevant MR. Furthermore, MR was not quantified by PISA/ERO and there was only a small number of 3D echocardiographies. We acknowledge the fact that our study endpoint of redo MV surgery potentially misses those patients who develop significant MR after MV repair, but are never referred for redo surgery. Potential reasons for nonreferring these patients for redo surgery may include (1) rejection due to prohibitive surgical risk, (2) patients refuse redo surgery themselves, (3) death before diagnosis, or (4) physicians/cardiologists are reluctant to refer the patient for redo surgery despite relevant MR. Furthermore some patients may have been transferred to other institutions for redo surgery and therefore not captured by our database. Given these limitations, a systematic echocardiographic follow-up of all patients who underwent previous MV repair is obligatory to obtain more sophisticated data on MR recurrence and was not carried out at our institution over the period of the last 11 years.

In addition, an observational study over a long time period may be potentially biased by continuous improvement in the surgical techniques over time and improving skills of the surgical team. 

## 6. Conclusions

Although rare, recurrent mitral valve regurgitation is still a significant problem and is associated with relevant perioperative mortality. Our study supports the fact that patients with severe MR should be admitted to early surgery as long as systolic pulmonary artery pressure is low and left ventricular ejection fraction is preserved.

## Figures and Tables

**Table 1:** Preoperative and operative characteristics and *p*-values for matching parameters. Echocardiographical data was collected before primary surgery and operative characteristics describe surgical strategy for primary repair.

	Whole Cohort (*n* = 215)	Redo Group (*n* = 77)	Control Group (*n* = 138)	*p*-Value for Matching
Age, y	61 ± 11	60 ± 11	61 ± 11	0.58
Male	131 (61%)	42 (55%)	89 (64%)	0.85
Preoperative echocardiographic assessment				
Ejection fraction, %				
preserved (m 52–72; f 54–74)	151 (71%)	49 (64%)	102 (76%)	0.07
mild dysfunction (m 41–51; f 41–53)	30 (14%)	10 (13%)	20 (14%)	0.64
moderate dysfunction (m & f 30–40)	20 (9%)	11 (14%)	9 (6%)	0.02
severe dysfunction (m & f < 30)	14 (6%)	7 (9%)	7 (4%)	0.09
Etiology				
degenerative	147 (68%)	42 (55%)	105 (76%)	0.3
-Type II	140 (65%)	38 (50%)	102 (74%)	
-Type IIIa	7 (3%)	4 (5%)	3 (2%)	
functional	68 (32%)	35 (45%)	33 (24%)	0.3
-Type I	28 (13%)	15 (19%)	13 (10%)	
-Type IIIb	40 (19%)	20 (26%)	20 (14%)	
Leaflet prolapse	140 (66%)	38 (49%)	102 (74%)	<0.01
none	28 (13%)	15 (20%)	13 (9%)	<0.01
AML	21 (10%)	9 (12%)	12 (9%)	0.36
PML	99 (47%)	20 (26%)	79 (57%)	<0.01
bileaflet	20 (9%)	4 (5%)	16 (12%)	0.17
Leaflet restriction	47 (22%)	24 (31%)	23 (17%)	0.02
Operative Characteristics				
Cardiopulmonary bypass time, min	163 (51; 412)	160 (51; 412)	165 (51; 314)	0.9
Isolated Mitral Valve Repair	165 (77%)	40 (52%)	125 (90.6%)	<0.01
Minimally invasive approach	133 (62%)	16 (21%)	117 (85%)	<0.01
Concomitant Procedures	50 (23%)	37 (48%)	13 (9%)	<0.01
CABG	20 (7%)	10 (13%)	10 (7%)	
AVR	13 (5%)	12 (15%)	1 (0.7%)	
CABG + AVR	1 (0.4%)	-	1 (0.7%)	
Tricuspid Valve Surgery	9 (4%)	8 (10%)	1 (0.7%)	
CABG + Tricuspid Valve Surgery	2 (0.7%)	2 (3%)	-	
AVR + Tricuspid Valve Surgery	1 (0.4%)	1 (1%)	-	
Ablation Procedure	3 (1.4%)	3 (4%)	-	
Septal Myectomy	1 (0.4%)	1 (1%)	-	
Size of mitral annuloplasty				
28–36 mm	205 (96%)	72 (93%)	133 (97%)	0.98
Type of leaflet repair				
Neochordae	36 (17%)	14 (18%)	22 (16%)	0.91
Resection	58 (27%)	7 (9%)	51 (37%)	0.008
Neochordae + Resection	17 (8%)	4 (5%)	13 (9%)	0.83

AML, anterior mitral leaflet; PML, posterior mitral leaflet; CABG, coronary artery bypass graft; AVR, aortic valve replacement.

**Table 2:** Echocardiographical risk factors for redo mitral valve (MV) surgery in the whole study cohort (Cox regression model). Parameters were collected prior to initial mitral valve repair.

Multivariate Analysis	Entire Study Cohort (*n* = 215)			
Variable	Redo Group	Control Group	HR	*p*-Value
Systolic PAP ≥ 50 mmHg	16 (21%)	13 (9%)	2.3	0.02
TR ≥ moderate	13 (17%)	14 (10%)	1.04	0.87
AML length (mm)	30 ± 8	30 ± 7	0.5	0.29
Pmean > 5 mmHg	7 (9%)	2 (1%)	3.3	0.001
LVEF ≤ 45%	15 (19%)	14 (10%)	2.1	0.05
LVEDD (mm)	62 ± 11	61 ± 9	2.3	0.78
MR at discharge ≥ moderate	17 (22%)	7 (5%)	5.8	0.002

MV, mitral valve; PAP, pulmonary artery pressure; TR, tricuspid regurgitation; AML, anterior mitral leaflet; Pmean, mean pressure gradient; LVEF, left ventricular ejection fraction; LVEDD, left ventricular enddiastolic diameter; MR, mitral regurgitation; HR, hazard ratio.

**Table 3:** Risk factors for redo MV surgery in the degenerative mitral regurgitation (DMR) subgroup (Cox regression model). Parameters were collected prior to initial mitral valve repair.

Multivariate Analysis	Degenerative Mitral Valve Disease (*n* = 147)			
Variable	Redo Group	Control Group	HR	*p*-Value
Bileaflet_prolapse	3 (8.3%)	17 (15%)	0.57	0.7
Non-PML prolapse	11 (31%)	30 (27%)	0.23	0.16
sPAP ≥ 50 mmHg	6 (17%)	9 (8%)	1.63	0.43
LVEF ≤ 45%	2 (5.5%)	3 (2.7%)	0.52	0.65
MR at discharge ≥moderate	7 (19.4%)	2 (1.8%)	14.5	0.006

MV, mitral valve; DMR, degenerative mitral regurgitation; PML, posterior mitral leaflet; PAP, pulmonary artery pressure; LVEF, left ventricular ejection fraction.

**Table 4:** Echocardiographical risk factors prior to initial mitral valve repair for redo MV surgery in the functional mitral regurgitation (FMR) subgroup (Cox regression model).

Multivariate Analysis	Functional Mitral Valve Disease (*n* = 68)			
Variable	Redo Group	Control Group	HR	*p*-Value
LVEDD ≥ 65 mm	13 (36%)	4 (12.5%)	3.67	0.043
PML angle (°)	35 ± 11	32 ± 12	0.2	0.49
AML length (mm)	28 ± 5	27 ± 7	0.01	0.48
Tenting height ≥ 8 mm	13 (36%)	6 (19%)	1.8	0.41
LVEF ≤ 45%	13 (36%)	11 (34%)	1.2	0.46
Parameters before discharge				
PML angle (°)	40 ± 12	40 ± 10	0.9	0.1
Tenting height ≥ 6 mm	11 (31%)	3 (9%)	2.3	0.018

MV, mitral valve; FMR, functional mitral regurgitation; LVEDD, left ventricular end-diastolic diameter; PML, posterior mitral leaflet; AML, anterior mitral leaflet; LVEF, left ventricular ejection fraction.

## Supplementary Materials

The following are available online at https://www.mdpi.com/2077-0383/8/4/526/s1, Video S1: Video 1 is an operative video showing papillary muscle repositioning, a novel technique to reduce incidence of failure after mitral valve repair in FMR patients with Type IIIb dysfunction.

## Abbreviations

MVR	mitral valve repair
MVD	mitral valve disease
MR	mitral regurgitation
LV	left ventricle
RV	right ventricle
LA	left atrium
FMR	functional mitral regurgitation
DMR	degenerative mitral regurgitation
TR	tricuspid regurgitation
LVEDD	left ventricular end-diastolic diameter
LVESD	left ventricular end-systolic diameter
AP	anteroposterior diameter of mitral annulus
AML	anterior mitral leaflet
PML	posterior mitral leaflet
EF	ejection fraction
sPAP	systolic pulmonary artery pressure
CBP	Cardiopulmonary Bypass
PTFE	polytetrafluoroethylene
AVR	Aortic valve replacement

## References

[1] Madesis A., Tsakiridis K., Zarogoulidis P., Katsikogiannis N., Machairiotis N., Kougioumtzi I., Kesisis G., Tsiouda T., Beleveslis T., Koletas A. (2014). Review of mitral valve insufficiency: Repair or replacement. J. Thorac. Dis..

[2] Nishimura R.A., Otto C.M., Bonow R.O., Carabello B.A., Erwin J.P., Fleisher L.A., Jneid H., Mack M.J., McLeod C.J., O’Gara P.T. (2017). 2017 AHA/ACC focused update of the 2014 AHA/ACC guideline for the management of patients with valvular heart disease: A report of the american college of cardiology/american heart association task force on clinical practice guidelines. J. Am. Coll. Cardiol..

[3] Coutinho G.F., Antunes M.J. (2017). Mitral valve repair for degenerative mitral valve disease: Surgical approach, patient selection and long-term outcomes. Heart.

[4] EACTS ESC/EACTS guidelines for the management of valvular heart disease published today. https://academic.oup.com/eurheartj/article/38/36/2739/4095039#114546712.

[5] Tatum J.M., Bowdish M.E., Mack W.J., Quinn A.M., Cohen R.G., Hackmann A.E., Barr M.L., Starnes V.A. (2017). Outcomes after mitral valve repair: A single-center 16-year experience. J. Thorac. Cardiovasc. Surg..

[6] Popovic Z.B., Sato K., Desai M.Y. (2018). Is universal grading of diastolic function by echocardiography feasible?. Cardiovasc. Diagn. Ther..

[7] Gillinov A.M., Cosgrove D.M. (2002). Mitral valve repair for degenerative disease. J. Heart Valve Dis..

[8] Adams D.H., Anyanwu A.C., Rahmanian P.B., Abascal V., Salzberg S.P., Filsoufi F. (2006). Large annuloplasty rings facilitate mitral valve repair in barlow’s disease. Ann. Thorac. Surg..

[9] Carpentier A., Adams D.H., Filsoufi F. (2010). Carpentier’s Reconstructive Valve Surgery: From Valve Analysis to Valve Reconstruction.

[10] Ciarka A., Braun J., Delgado V., Versteegh M., Boersma E., Klautz R., Dion R., Bax J.J., Van de Veire N. (2010). Predictors of mitral regurgitation recurrence in patients with heart failure undergoing mitral valve annuloplasty. Am. J. Cardiol..

[11] Castillo-Sang M., Guthrie T.J., Moon M.R., Lawton J.S., Maniar H.S., Damiano R.J., Silvestry S.C. (2015). Outcomes of repeat mitral valve surgery in patients with pulmonary hypertension. Innovations (Phila).

[12] Murashita T., Okada Y., Kanemitsu H., Fukunaga N., Konishi Y., Nakamura K., Koyama T. (2015). The impact of preoperative and postoperative pulmonary hypertension on long-term surgical outcome after mitral valve repair for degenerative mitral regurgitation. Ann. Thorac. Cardiovasc. Surg..

[13] Chenot F., Montant P., Vancraeynest D., Pasquet A., Gerber B., Noirhomme P.H., El Khoury G., Vanoverschelde J.L. (2009). Long-term clinical outcome of mitral valve repair in asymptomatic severe mitral regurgitation. Eur. J. Cardiothorac. Surg..

[14] Wang J., Han J., Li Y., Xu C., Jiao Y., Yang B., Meng X., Bolling S.F. (2014). Preoperative risk factors of medium-term mitral valve repair outcome. Interact. Cardiovasc. Thorac. Surg..

[15] Zhuo D.X., Bilchick K.C., Mazimba S. (2018). Preoperative invasive hemodynamic determinants of survival among patients undergoing aortic or mitral valve surgery. J. Cardiothorac. Vasc. Anesth..

[16] Pardi M.M., Pomerantzeff P.M.A., Sampaio R.O., Abduch M.C., Brandao C.M.A., Mathias W., Grinberg M., Tarasoutchi F., Vieira M.L.C. (2018). Relation of mitral valve morphology to surgical repair results in patients with mitral valve prolapse: A three-dimensional transesophageal echocardiography study. Echocardiography.

[17] Ghoreishi M., Evans C.F., DeFilippi C.R., Hobbs G., Young C.A., Griffith B.P., Gammie J.S. (2011). Pulmonary hypertension adversely affects short- and long-term survival after mitral valve operation for mitral regurgitation: Implications for timing of surgery. J. Thorac. Cardiovasc. Surg..

[18] Coutinho G.F., Correia P.M., Branco C., Antunes M.J. (2016). Long-term results of mitral valve surgery for degenerative anterior leaflet or bileaflet prolapse: Analysis of negative factors for repair, early and late failures, and survival. Eur. J. Cardiothorac. Surg..

[19] David T.E., Armstrong S., McCrindle B.W., Manlhiot C. (2013). Late outcomes of mitral valve repair for mitral regurgitation due to degenerative disease. Circulation.

[20] Shimokawa T., Kasegawa H., Katayama Y., Matsuyama S., Manabe S., Tabata M., Fukui T., Takanashi S. (2011). Mechanisms of recurrent regurgitation after valve repair for prolapsed mitral valve disease. Ann. Thorac. Surg..

[21] Suri R.M., Clavel M.A., Schaff H.V., Michelena H.I., Huebner M., Nishimura R.A., Enriquez-Sarano M. (2016). Effect of recurrent mitral regurgitation following degenerative mitral valve repair: Long-term analysis of competing outcomes. J. Am. Coll. Cardiol..

[22] Gelsomino S., Lorusso R., Caciolli S., Capecchi I., Rostagno C., Chioccioli M., De Cicco G., Bille G., Stefano P., Gensini G.F. (2008). Insights on left ventricular and valvular mechanisms of recurrent ischemic mitral regurgitation after restrictive annuloplasty and coronary artery bypass grafting. J. Thorac. Cardiovasc. Surg..

[23] Hung J., Papakostas L., Tahta S.A., Hardy B.G., Bollen B.A., Duran C.M., Levine R.A. (2004). Mechanism of recurrent ischemic mitral regurgitation after annuloplasty: Continued lv remodeling as a moving target. Circulation.

[24] Kron I.L., Hung J., Overbey J.R., Bouchard D., Gelijns A.C., Moskowitz A.J., Voisine P., O’Gara P.T., Argenziano M., Michler R.E. (2015). Predicting recurrent mitral regurgitation after mitral valve repair for severe ischemic mitral regurgitation. J. Thorac. Cardiovasc. Surg..

[25] Acker M.A., Parides M.K., Perrault L.P., Moskowitz A.J., Gelijns A.C., Voisine P., Smith P.K., Hung J.W., Blackstone E.H., Puskas J.D. (2014). Mitral-valve repair versus replacement for severe ischemic mitral regurgitation. N. Engl. J. Med..

[26] McGee E.C., Gillinov A.M., Blackstone E.H., Rajeswaran J., Cohen G., Najam F., Shiota T., Sabik J.F., Lytle B.W., McCarthy P.M. (2004). Recurrent mitral regurgitation after annuloplasty for functional ischemic mitral regurgitation. J. Thorac. Cardiovasc. Surg..

[27] Roshanali F., Mandegar M.H., Yousefnia M.A., Rayatzadeh H., Alaeddini F. (2007). A prospective study of predicting factors in ischemic mitral regurgitation recurrence after ring annuloplasty. Ann. Thorac. Surg..

[28] Goldstein D., Moskowitz A.J., Gelijns A.C., Ailawadi G., Parides M.K., Perrault L.P., Hung J.W., Voisine P., Dagenais F., Gillinov A.M. (2016). Two-year outcomes of surgical treatment of severe ischemic mitral regurgitation. N. Engl. J. Med..

[29] Kron I.L., Green G.R., Cope J.T. (2002). Surgical relocation of the posterior papillary muscle in chronic ischemic mitral regurgitation. Ann. Thorac. Surg..

[30] Harmel E.K., Reichenspurner H., Girdauskas E. (2018). Subannular reconstruction in secondary mitral regurgitation: A meta-analysis. Heart.

[31] Nappi F., Spadaccio C., Nenna A., Lusini M., Fraldi M., Acar C., Chello M. (2017). Is subvalvular repair worthwhile in severe ischemic mitral regurgitation? Subanalysis of the papillary muscle approximation trial. J. Thorac. Cardiovasc. Surg..

[32] Misumi Y., Masai T., Toda K., Nakamura T., Miyagawa S., Yoshikawa Y., Fukushima S., Saito S., Domae K., Kainuma S. (2017). Restrictive mitral annuloplasty with or without papillary muscle approximation for functional mitral regurgitation. J. Heart Valve Dis..

